# The Kansas Medical Society

**Published:** 1877-07

**Authors:** 


### THE KANSAS MEDICAL SOCIETY

met in Lawrence, May 9th, and was called to order by the President, Dr. H. S. Roberts, of Manhattan; Dr. D. C. Jones, of Topeka, was elected Secretary *pro tem.*

The reading of the minutes of the last annual meeting was dispensed with, and the minutes, as published, were adopted.

The President read his annual address, which was referred to the committee on publication.

A motion to so amend the by-laws as to require graduation as a qualification for membership, was offered and adopted.

Dr. Mottram, of Lawrence, made a report from the committee on surgery, and Dr. Shoyer, of Leavenworth, from the committee on obstetrics. The reports were referred to the committee on publication.

Dr. Schenck, of Osage City, reported on septic diseases. The report was very favorably received, discussed and ordered published.

### SECOND DAY.

This Society convened again this morning at 8 o'clock, and was called to order by the President. About forty members were present.

Dr. Todd, of Kansas City, delivered a lecture on Obstetrics, after which the various reports were heard. The committee on necrology on the death of Dr. J. S. Redfield, of Fort Scott. Report on typhoid fever, by Dr. Van Eman, of Tonganoxie; on syphilis, by Dr. Cochran, of Atchison. Voluntary papers, by Dr. Halley, of Kansas City; Dr. Hanawalt, of Arvonia; Dr. Morris, of Lawrence; and Dr. Lawrence, of Emporia, and all appropriately referred.

The election of officers for the ensuing year resulted as follows:

President, W. L. Schenck, of Osage City; 1st Vice-President, C. C. Furley, of Wichita; 2d Vice-President, J. H. Stuart, of Lawrence; Secretary, F. D. Moss, of Lawrence; Assistant Secretary, V. Biart, of Leavenworth; Treasurer, W. W. Cochran, of Atchison; Censors, H. O. Hanawalt, Arvonia; H. S. Roberts, Manhattan; G. R. Baldwin, Fort Scott; G. L. Sears, Atchison; P. O'Brien, Topeka.

The Society then adjourned to meet at Topeka on the second Wednesday of May, 1878.

